# Serum levels of Selenium and C-reactive protein in comatose patients with severe traumatic brain injury during the first week of hospitalization: case-control study

**DOI:** 10.11604/pamj.2018.29.36.10945

**Published:** 2018-01-16

**Authors:** Bahia Belatar, Fatna Laidi, Abdelah El Abidi, Rachid Eljaoudi, Fouzia Mamouch, Saad Kabbaj, Wajdi Maazouzi

**Affiliations:** 1 Research Unit of Cerebral Monitoring in Neuro-reanimation, Faculty of Medicine and Pharmacy, University Mohammed V of Rabat, Morocco; 2 Oral Biomechanics and Biotechnology Research Unit, Faculty of Dental Medicine, Faculty of Medicine and Pharmacy, University Mohammed V of Rabat, Morocco; 3 Department of Toxicology, National Institute of Health, Rabat, Morocco; 4 Pharmacology and Toxicology Department, Faculty of Medicine and Pharmacy, University Mohammed V of Rabat, Morocco; 5 Research Unit of Oncology, Faculty of Medicine and Pharmacy, University Mohammed V of Rabat, Morocco; 6 Service of Anesthesiology and Reanimation, Hospital of Specialties, Ibn Sina University Hospital, Morocco

**Keywords:** Severe brain injury, selenium, coma, C-reactive protein, comatose patients, serum selenium, serum C-reactive protein

## Abstract

**Introduction:**

Mortality and morbidity related to traumatic brain injuries still remain high in patients. Many authors reported the importance of Selenium in maintaining the integrity of brain functions. This fact is supported by clinical evidence that therapy with selenium supplementation could help patients suffering from brain disorders like neurodegenerative diseases. The aim of our study was to assess the relationship between Selenium concentration in serum and evolution of comatose patients with severe traumatic brain injury, in the first week of admission, and the correlation between selenium and C-reactive protein.

**Methods:**

This case-control study was conducted with 64 comatose patients with TBI, in the Department of Anesthesiology and Reanimation, IbnSina University Hospital and Hospital of specialties in Rabat-Morocco, and healthy volunteers recruited in Blood transfusion center of Rabat. Blood sampling was collected from TBI patients, in the first week (3h after admission and each 48h during one week), and from healthy volunteers one time. Concentration of Se in serum was determined by electrochemical atomic absorption spectrometry. Statistical analysis was performed using Statistical software (SPSS) and the cases and controls were compared using the Mann-Whitney U test. A P-value < 0.05 was considered to be statistically significant.

**Results:**

Comparison selenium concentration in the first day (D0), third day (D2) and fifth day according to the death and survival statue in patients did not show statistical significance (p > 0.05). Selenium concentration of D0 in patients and Selenium concentration in control group also did not show statistical significance (p > 0.05). Similarly, we did not report a correlation between selenium and C-reactive protein.

**Conclusion:**

According to our data selenium and CRP may not play a role in progression of coma state in patients with severe traumatic brain injury.

## Introduction

Selenium (Se) is generally known as an essential micronutrient and antioxidant for humans and animals [1].This element is an essential component of a number of selenoproteins including the glutathione peroxidase which is a family of enzymes that protect against oxidative injury by catalyzing the breakdown of hydrogen peroxide and lipid hydroperoxides [2]. Selenium is incorporated into 25 selenoproteins with different activities including protection against lipid peroxidation, thyroid hormone metabolism, T cell immunity and modulation of inflammatory response [3]. The main selenoproteins in blood are selenoproteins P which contribute approximately 50% of the total plasmatic selenium and glutathione peroxidase which count 10-30% of selenium in plasma [4]. In the brain, selenium concentration is reported to be far less variable than those in the peripheral tissues and organs [5]. This fact implied the importance of selenium for maintaining the integrity of brain functions and the distinctive selenium metabolism and the regulatory mechanism in the brain [5]. Otherwise, studying the relationship between selenium and progression of patients suffering from brain disorder such as traumatic brain injury (TBI) may be useful. In fact, the Mortality and morbidity related to traumatic brain injuries still remain high in patients [6]. Some studies had reported positive clinical responses obtained during therapy with selenium and other antioxidants in neurodegenerative diseases [7]. Similarly, neuroprotective effects of Se have been reported [8]. Selenium is essential for the formation of selenoproteins, particularly in the central nervous system [9]. The C-reactive protein is another protein reported as a robust biomarker to predict secondary pathologies associated with TBI [10]. In fact, serum CRP level is strongly correlated to severity of the brain injury in patients [10]. This protein is known as a sensitive biomarker of systemic inflammation in response to infection, trauma, surgery, burns, tissue infarction, advanced cancer, and chronic inflammatory conditions [11, 12]. In association with selenium, some authors reported an inverse correlation between selenium and C-reactive protein in patients with respiratory diseases [13]. The aim of our study was to assess the relationship between Selenium concentration in serum and evolution of comatose patients with severe traumatic brain injury, within the first week of admission, and the correlation between selenium and C-reactive protein.

## Methods

**Study design:** This case-control study was conducted with 64 comatose patients with TBI identified as cases and healthy volunteers identified as controls recruited in Blood transfusion center of Rabat.

### Selection criteria of cases and controls

**Comatose patients with Traumatic brain injury:** Patients included in this study were recruited during the period between December 2013 and January 2015 in the Department of Anesthesiology and Reanimation, IbnSina University Hospital and Hospital of specialties in Rabat, Morocco. We studied 65 comatose patients with severe Traumatic Brain Injury (TBI). 57 men aged 42.7±13.8 years old and 7 women aged 39.00 ±17.71 years old.

**Inclusion criteria:** Cases included in this study were comatose patients with severe TBI aged from 18 to 64 years old. Neurological symptoms of severe TBI were confirmed by MRI/CT-scan.

**Exclusion criteria:** Patients excluded from the study were smoker, alcoholic, receiving barbiturates medication, corticoids or any treatment that could have an antioxidant effects and patients who underwent a craniotomy, with febrile state and/or septic shock state and psychological problems.

**Healthy volunteers:** Healthy volunteers did not show clinical symptoms or sign of any diseases. All participants were mentally and physically able to participate in the study. The age of volunteers was matched with the age of patients: 30 men aged 33.53±8.76 years old and 23 women aged 39.78±12.34 years old were included. The study protocol was approved by a local Ethics Committee for Biomedical Research in Rabat, Morocco. Healthy volunteers have read and signed a consent form before to be included in the study.

**Blood sampling and storing conditions:** 4ml of Peripheral venous blood was collected in serum tube. Blood sampling in TBI patients was performed within the first week (3h after admission and each 48h during one week). Blood samples were centrifuged at 4.000rpm/min for 15 min at 4°C. Then serum samples were aliquoted and stored at -80°C for later determination of selenium.

**Laboratory test:** We determined the concentration of Se in serum by electrochemical atomic absorption spectrometry (VARIAN AA 240 atomic absorption spectrophotometer equipped with Zeeman background corrector), with graphite furnace. A selenium electrode less discharged lamp, HGA (graphite tube) and Argon (inert gas) were used. All analyses were performed at 196.0 nm wavelength and 2.0 nm slit width. The concentration of CRP and creatinine were collected from medical records and compared with selenium concentrations.

**Statistical analyses:** The statistical analysis was performed using Statistical Package for the Social Sciences version 13.0 software (SPSS Inc., Chicago, IL, USA). The concentration of Se, CRP and creatinine were expressed in median and interquartile, the age was expressed in mean and standard deviation. The cases and controls were compared using the Mann-Whitney U test. A P-value <0.05 was considered to be statistically significant.

## Results

**Figure 1:**
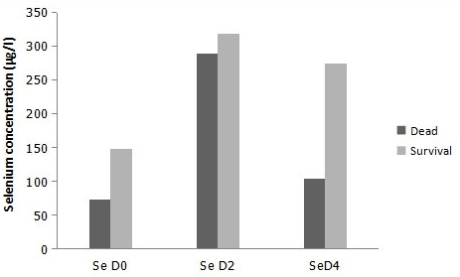
Concentration of Selenium D0, D2 and D4 according to death and survival statue of patients (p = 0.95)

**Figure 2:**
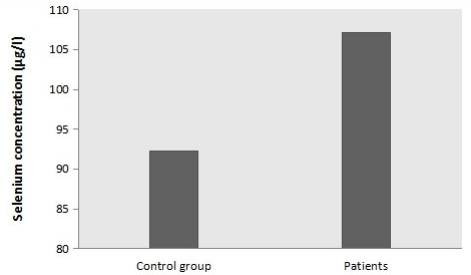
Concentration of Selenium D0 in patients and controls (p = 0.74)

Clinical data are summarized in Table 1. Indeed, most of traumatic brain injury cases received in the hospital were men (89.1%) and only 10.9% of patients were women. Our data has shown that the main type of severe traumatic brain injury was Brain injury type (71.9%). The other types of severe TBI were respectively 21.9% and 6.3% for Hemorrhagic stroke and ischemic stroke. The Death rate in patients was relatively high (60.9%) compared to survival rate which was 39.1% only. The Glasgow Coma Scale scores were collected in three days within admission. In fact, all patients in the first day showed severe score of GCS. In the third day after admission, 90.9% had severe GCS score compared to 9.1% of moderate scores. In the fifth day GCS score were also 81.81% severe and 18.19% moderate. Comparison of age according to the death and survival rates in patients did not show statistical significance (p>0.05). The same data was remarked in the other serological tests. Comparison of C-reactive protein concentration, selenium in the first day (D0), third day (D2) and fifth day (D4) (Figure 1), in addition to concentration creatinine did not show statistical significance (p>0.05) (Table 2). We have compared the concentration of Creatinine and Selenium (D0, D2, and D4) in patients by gender in addition to Selenium concentration in control group also by gender (Table 3). Our data has shown that CRT concentration in D0 and D2 was higher in men (p=0.046 and p=0.002). However, CRT concentration in D4 did not show statistical significance between men and women (p>0.05). The same data was registered in Selenium concentration in D0, D2 and D4 in patients and control group according to gender (not statistically significant p>0.05). We have also studied the correlation between many quantitative parameters in patients with Severe Traumatic Brain Injury (Table 4). Correlation between Selenium in D2 and the age of patients was statistically significant (p=0.01). However, the coefficient of correlation found is weak (r=0.15). The same data was remarked in Selenium concentration of D4 and the age of patients: p=0.03 (statistically significant) the correlation was positive but weak (r=0.075). The other studied correlations (Selenium D0 and patient's age, Selenium D0 and Creatinine D0, Selenium D0 and C-reactive protein) were not statistically significant (p=0.05). Selenium concentration of D0 in patients and Selenium concentration in control group did not show statistical significance (p>0.05) (Figure 2).

**Table 1:** Clinical data collected from medical records of patients with Severe Traumatic Brain Injury within one week in admission

Clinical data of patients	
**Sexpercentage (%)**	
Female (n=7)	10.9
Male (n=57)	89.1
**Percentage (%) of severe TBI Types (n=64)**	
- IS (n=4)	6.3
- HS (n=14)	21.9
- BI (n=46)	71.9
**Death rate within one week (%) (n=39)**	60.9
**Survival rate within one week (%) (n=25)**	39.1
**GSC scores (%)**	
**D0 Severe (n=51)**	100
**D2**	
Severe (n=20)	90.9
Moderate (n=2)	9.1
**D4**	
Severe (n=9)	81.81
Moderate (n=2)	18.19

Abbreviations: **TBI:** traumatic brain injury; **IS:** ischemic stroke; **HS:** Hemorrhagic stroke; **BI:** brain injury; **GCS:** Glasgow Coma Scale. **D0:** first day; **D2:** third day; **D4:** fifth day.

**Table 2:** Comparison of serological tests in patients with Severe Traumatic Brain Injury according to the death and survival state of patients

Variables /Patients	Dead patients	Survived patients	P value
**Age**	46.03±13.63 (n=39)	36.48±13.33 (n=25)	0.09
CRP D0 (mg/l)	29.10 (2.30 ; 93.10) (n=7)	43.50 (2.70 ; 69.28) (n=11)	0.84
Se D0 (µg/l)	73.2 (5.65 ; 314.15) (n=30)	147.94 (34.72 ; 408.6) (n=15)	0.43
Se D2 (µg/l)	289.2 (54.84 ; 537.44) (n=19)	318.9 (28.67 ; 730.7) (n=12)	0.68
Se D4 (µg/l)	104.8 (0 ; 173.68) (n=9)	274.3 (85 ; 494.5) (n=9)	0.1
CRT D0 (mg/l)	7.9 (6.5; 13.6) (n=31)	7(5.45; 8.6) (n=21)	0.13
CRT D2 (mg/l)	8.65 (6.57; 11.45) (n=14)	8.9 (5.85 ; 8.9) (n=9)	0.68
CRT D4 (mg/l)	6.85 (5.6 ; 8.25) (n=8)	7.3 (5.45 ; 64) (n=5)	0.65

**Abbreviations: CRP:** C-reactiveprotein; **Se:** Selenium; **CRT:** creatinine

**Table 3:** Quantitative characteristics of the studied population by gender in patients and healthy volunteers

	Men	Women	P value
CRT D0 (mg/l)	7.8 (6.45; 13.75) (n = 45)	5.9 (4.5; 7.7) (n = 7)	0.046
CRT D2 (mg/l)	8.9 (7.1; 11.2) (n = 19)	5.8 (5.1; 5.87) (n = 4)	0.002
CRT D4 (mg/l)	7.3 (5.9; 8.1) (n = 9)	34.7 (5.32; 64) (n = 4)	0.81
Se D0 (µg/l) patients	87.68 (22.44; 249.92) (n = 55)	101(47.96; 159.56) (n = 15)	0.63
Se D2 (µg/l) patients	318.9 (82.57 ; 623.71) (n = 26)	102.4 (8.95; 761.52) (n = 5)	0.85
Se D4 (µg/l) patients	124.7 (75.65; 316.18) (n = 14)	160.99 (21.25; 772.19) (n = 4)	0.95
Se (µg/l) Control group	57.06 (18.36; 188.3) (n = 19)	99.4 (37.86; 180.91) (n = 17)	0.95

**Table 4:** Correlation between quantitative parameters in patients with Severe Traumatic Brain Injury

Variables		R (correlation coefficient)	P value
Se D0 in patients (n=44)	Patients Age	-	0.08
Se D2 in patients (n=33)	Patients Age	0.15	**0.01**
Se D4 in patients (n=33)	Patients Age	0.075	**0.03**
Se D0 in patients (n=27)	CRT D0 (mg/l)	-	0.27
Se D0 in patients (n=45)	CRP (mg/l) (n=10)	-	0.21

## Discussion

Selenium was found to be implied in many diseases such as rheumatoid arthritis, lung cancer,adultT-cell leukemia [14]. Indeed a lower selenium level was found in patient with all described illnesses compared to healthy subjects [14]. Some authors reported that Selenium concentration in blood decreases according to the magnitude of the inflammatory response [15]. Similarly, other authors finding revealed that supplementation with selenium had an immune-stimulant effects including an improvement of proliferation of activated T cells [16]. As regards TBI patients, our data may suggest that selenium may not play a role in death of patients with TBI. Selenium levels were not higher in patients who died from a TBI compared to patients who survived after TBI. Moreover, the difference of selenium level in patients with TBI and healthy subjects was not significant. Otherwise, Selenium was also studied with C-reactive protein in many illnesses and the results demonstrated an important role of C-reactive protein in the acute phase response in several ones such as rheumatoid arthritis, lung cancer, adult T-cell leukemia [14]. In fact, the level of CRP was higher in patients with different diseases compared to healthy subjects [14]. The C-reactive protein was widely reported as a marker for underlying systemic inflammation [17]. Furthermore, a strong correlation between serum CRP level and severity of brain injury in patients was also reported [10]. Besides, selenium and CRP serum levels were found to be related to age and gender in healthy subjects [14]. In contrast, our data did not show any significance of selenium concentration (D0, D2 and D4) in patients according to gender. Selenium concentration in the human body was found to vary with age [18]. For instance, selenium concentration in fetal brain decreased with age, but increased with age post natally [19]. Our data showed a positive and weak correlation between Selenium in D2 and the age of patients same as Selenium concentration of D4 and the age of patients. The other studied correlations (Selenium D0 and patient's age, Selenium D0 and Creatinine D0, Selenium D0 and C-reactive protein) were not statistically significant. From the literature point of view, an inverse correlation between selenium and C-reactive protein in patients with respiratory diseases was found [13]. However the correlation was weak [13]. A low plasma concentration of selenium and high concentration of CRP in septic patients within admission was remarked [20]. However the correlation between selenium and CRP was not significant [20]. In healthy elders, blood selenium levels were negatively correlated with age [21]. Serum concentration of Se in healthy subjects was higher than in patients with Ischemic stroke and the difference was statistically significant [22]. Other prognostic factors of TBI (age and serum creatinine concentration) were studied in patients according to death and survival statue, the results may suggest that age and creatinine levels were not implied in patient's death. This data is supported by similar study: no significance of serum creatinine levels in children according to survival and non-survival statue after Traumatic Brain injury [23]. However in patients, we remarked a higher serum creatinine level in D0, D2 for men. Although the difference of creatinine level in D4 did not show difference according to gender of patients.

## Conclusion

According to our data selenium and CRP may not play a role in progression of coma state in patients with severe traumatic brain injury.

### What is known about this topic

- Selenium is an important element for maintaining the integrity of brain functions. The C-reactive protein is known as a robust biomarker to predict secondary pathologies associated with traumatic brain injury;
- Therapy supplementation with selenium had proved efficiency in some neurodegenerative diseases;
- A negative association between selenium and C-reactive protein was found in some illnesses including inflammatory and respiratory diseases.

### What this study adds

- A lack of selenium concentration in blood may not play a role in dead of patients after a severe traumatic brain injury;
- The concentration of serum C-reactive protein is not correlated to selenium concentration in blood;
- The concentration of selenium is weakly correlated to the age of patients in contrast to similar studies.

## Competing interests

The authors declare no competing interests.
